# Executive Dysfunction and Reduced Self-Awareness in Patients With Neurological Disorders. A Mini-Review

**DOI:** 10.3389/fpsyg.2020.01697

**Published:** 2020-07-14

**Authors:** Martina Amanzio, Massimo Bartoli, Giuseppina Elena Cipriani, Sara Palermo

**Affiliations:** ^1^Department of Psychology, University of Turin, Turin, Italy; ^2^European Innovation Partnership on Active and Healthy Ageing, Brussels, Belgium

**Keywords:** reduced self-awareness, Alzheimer’s disease, behavioural Frontotemporal Dementia, Acquired Brain Injury, executive functions, Cognitive Awareness Model

## Abstract

Awareness of deficits in patients with neurological disorders may be described as a theoretical unitary phenomenon, which has been analysed reaching interesting results in the last decades. Awareness of deficits manifests itself in a continuum ranging from full awareness to total absence. In line with a neurocognitive approach, a reduction in self-awareness could be explained considering executive dysfunction associated with prefrontal cortex anatomo-functional changes. Our mini-review will focus on reduced self-awareness in neurological disorders, such as Alzheimer’s disease, behavioural Frontotemporal Dementia and Acquired Brain Injuries. Results achieved thanks to an explanatory investigative approach combined with a theoretical reference model will be presented. Data suggest the key role of executive functions in supporting adequate self-awareness towards patients’ cognitive-behavioural profile and instrumental activity autonomy. The Cognitive Awareness Model seems to be one of the best theoretical model to better approach this phenomenon.

## Introduction

Awareness of deficits appears in a continuum ranging from full awareness to total absence. In patients with neurological disorders, it may be described as a theoretical unitary phenomenon, which has been analysed reaching interesting results in the last two decades. A reduction in self-awareness could be explained considering both prefrontal cortex anatomo-functional changes and executive dysfunction in patients with Alzheimer’s disease (AD), behavioural Frontotemporal Dementia (bvFTD) and Acquired Brain Injury (ABI) (Amanzio et al., 2011, 2013, 2016, 2017; Palermo et al., 2014). Indeed, executive functions are important in supporting adequate self-awareness with respect to the cognitive-behavioural framework and instrumental activities of daily living (IADL) autonomy (Amanzio et al., 2016, 2018). O’Keeffe et al. (2007) suggested that deficits in some executive functions–monitoring, response inhibition and cognitive flexibility–might affect patients’ judgment in terms of reduced self-awareness.

In our opinion, this phenomenon could be accurately described only by adopting a neurocognitive approach as a theoretical framework. In particular, this perspective allows to estimate neuroimaging anatomical-functional data and neuropsychological evidence in an unicum, considering the role of executive dysfunction in reducing self-awareness.

Our mini-review we will focus on reduced self-awareness in neurological disorders, such as Alzheimer’s disease (AD), behavioural Frontotemporal Dementia (bvFTD), and Acquired Brain Injury (ABI).

## Reduced Self-Awareness and Executive Functions: A Neurocognitive Theoretical Framework

**FIGURE 1:**
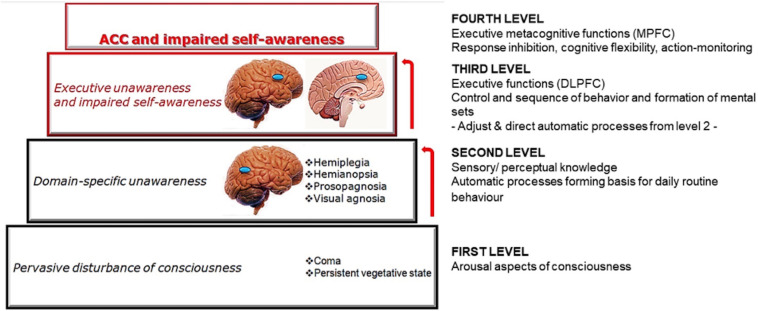
A graphical representation of the model proposed by Stuss et al. (2001) and Stuss and Anderson (2004). The figure describes input information processing starting from the sensory/perceptual knowledge level up to basic EFs and executive-metacognitive functions that pertain to the MPFC, with its core hub in MCC.

The neurocognitive approach emphasises the association between reduced awareness and brain diseases. In particular, it takes into account focal lesions, cognitive deficits, and motivational and emotional aspects (McGlynn and Schacter, 1989). Considering patients affected by neurological disorders with a reduced self-awareness, the model proposed by Stuss et al. (2001) and Stuss and Anderson (2004) represents a fundamental first theoretical background to understand how different awareness deficits corresponds to different brain circuitry. The authors hypothesised four distinct hierarchical levels referring to cortico-subcortical anatomical areas, where higher levels use the modelling abilities of the lower ones (see Figure 1).

Considering this hierarchy, Clare et al. (2011) conceived a “levels of awareness framework” characterised by different grades of awareness, following a progressive complexity. This multidimensional model pays attention not only to cognitive aspects but also to psychosocial and environmental impacts. Thus, it could be useful in order to develop personal and individual interventions (Sunderaraman and Cosentino, 2017).

In the model proposed by Stuss et al. (2001) and Stuss and Anderson (2004), information follow a bottom-up pathway, which terminates activating generator neurons al high levels. If a functional domain is impaired, a consciousness/awareness deficit could be present. In fact, we can observe a dissociation among different factors of functional domains.

Our mini-review takes into consideration only the highest level of conscious processing, which characterises self-awareness disturbances related to executive dysfunction.

There is an anatomical-functional specificity of the prefrontal regions of the brain. In particular, there are two main areas: the dorsolateral prefrontal cortex (DLPFC) and the medial prefrontal cortex (MPFC)– which represent, respectively, the third and fourth levels of the hierarchical model (Stuss et al., 2001; Stuss and Anderson, 2004). From a neuropsychological perspective, the DLPFC is involved in the control and sequencing of behaviour, and in the formation of mental sets, while the MPFC is implicated in metacognitive-executive functions, drive and motivation (Figure 1).

At this highest level, identifying an association between a reduction of self-awareness and executive dysfunctions could represent an adequate method to better understand the phenomenon. Executive models of self-awareness have been developed focusing on the damages of hypothetical higher order executive processes of control and action, which seem to play a key role in self-monitoring-action-abilities (Schacter, 1990; Stuss and Levine, 2002). In particular, Self-awareness relates to different metacognitive abilities: recognition of difficulties, understanding cognitive and functional consequences of deficits, achieving aims, and preventing possible issues disease-related (Ownsworth et al., 2007, 2008), mainly described in patients with traumatic brain injury and ABI (Ownsworth and Clare, 2006; Ownsworth et al., 2008).

Executive functions (EFs) refer to a cognitive domain holding various competences (Stuss and Benson, 1986). In the literature, among different EFs models, Latzman and Markon (2010) developed a structure, through the scores on the Delis-Kaplan Executive Function System (D-KEFS), which includes three factors: monitoring, response inhibition, and conceptual flexibility.

Also Miyake et al. (2000) presented a similar model composed by three basic EFs: “updating-monitoring,” “inhibition,” and “shifting.” Interestingly, they can be summarised and defined as “metacognition,” i.e., the capacity to control and estimate their own performance and knowledge (Flavell, 1979; Brown, 1980). Metacognition refers also to the ability to direct their own behaviour. Specifically, it concerns the capacity to regulate cognition and action in various social settings (Fernandez-Duque et al., 2000), which are closely related to DLPFC functioning (Fernandez-Duque et al., 2000; Shimamura, 2000; Fleming and Dolan, 2012; Frith, 2012). Particularly, we can observe two different pathways of the right DLPFC, providing evidence for the existence of an anterior DLPFC-anterior cingulate cortex (ACC) and a DLPFC-posterior parietal circuit (Cieslik et al., 2013). These networks are hierarchically structured: the posterior area seems to be related to the monitoring of action, more involved in stimulus processing and working memory; the anterior DLPFC-ACC network, instead, seems to be implicated with the higher-order-control-processes of motor behaviour, important in cognitive set-shifting and in inhibition of responses to insignificant stimuli (Cieslik et al., 2013).

Considering the above, we suggest to understand the role of EF in reduced self-awareness by taking into account basic EFs (Miyake et al., 2000). Importantly, specific subcomponents of executive-metacognitive functions—such as response inhibition, cognitive flexibility and action-monitoring—had been previously studied and associated with a reduction in self-awareness through a neurocognitive approach not only in neurodegenerative disorders such as AD and bvFTD, but also in Parkinson’s Disease (Amanzio et al., 2010, 2014; Palermo et al., 2017, 2018).

The *Cognitive Awareness Model* (CAM) (Agnew and Morris, 1998; Mograbi and Morris, 2014), originally developed to explain awareness deficits in AD, may be applied in both neurodegenerative disorders (O’Keeffe et al., 2007) and ABI patients (Sherer et al., 1998; Prigatano, 1999; Ownsworth et al., 2002). This model is characterised by a comparator system in the central executive system, which controls mismatches between a personal database and consciousness of failure in cognitive or in behavioural performances (Agnew and Morris, 1998; Mograbi and Morris, 2014). When a mismatch is detected, a signal is sent to the *Metacognitive Awareness System* (MAS) – the fourth level in the model proposed by Stuss et al. (2001) and Stuss and Anderson (2004) – causing consciousness of failure. which If the executive system is impaired, the comparator mechanism will not detect mismatches. Consequently, failures in cognitive performance will not reach metacognitive awareness.

Importantly, it had been proposed that the central executive system (Baddeley, 1986) accounts for a reduction of awareness in AD (Lopez et al., 1994), in particular when it is severely impaired.

## Selection of Studies

Studies on self-awareness, published from 30th April 2000 until 30th April 2020, were identified by a selection strategy across the online international database (Medline database with PubMed literature search). We used a single set of query terms: “reduced awareness” combined with pathology. Only relevant literature on neurological patients on AD, bvFTD and ABI was considered. Inclusion criteria contemplate original studies using structural or functional MRI and/or neuropsychological assessment.

The complete list of articles identified through research and the selection process are presented in Table 1.

**TABLE 1:** Synopsis of the studies selection.

	Pubmed ID	First author (year of publication)	Inclusion/exclusion (with reasons)	Methods (neurpsychological/neuroimaging)	Awareness assessment
Alzheimer	10733015	Robinson (2000)	Excluded (No neuropsychological or neuroimaging evaluation)		
	18161073	Bonney et al. (2007)	Included	Neuropsychological assessment	Dysexecutive Questionnaire (DEX, Wilson et al., 1996)
	20808100	Orfei et al. (2010)	Included	Neuropsychological assessment	Anosognosia Questionnaire for Dementia (AQ-D, Migliorelli et al., 1995) Clinical Insight Rating Scale (CIRS, Ott et al., 1996)
	20921874	Greenop et al. (2011)	Excluded (No diagnosis of AD or MCI likely due to AD)		
	21385751	Amanzio et al. (2011)	Included	Functional MRI and neuropsychological assessment	Anosognosia Questionnaire for Dementia (AQ-D, Migliorelli et al., 1995)
	21495076	Galeone et al. (2011)	Included	Neuropsychological assessment	Questionnaire based on patients and caregivers discrepancy score [adapted from Ansell and Bucks (2006)].
	22697174	Brookes et al. (2013)	Excluded (No diagnosis of AD or MCI likely due to AD)		
	22995647	Amanzio et al. (2013)	Included	Neuropsychological assessment	Anosognosia Questionnaire for Dementia (AQ-D, Migliorelli et al., 1995)
	25481475	Spalletta et al. (2014)	Included	Structural MRI and neuropsychological assessment	Memory Insight Questionnaire (MIQ, Marková et al., 2004)
	26385947	Tonga et al. (2016)	Excluded (No neuropsychological or neuroimaging evaluation)		
	27534380 28633865	Amanzio et al. (2016)Amanzio et al. (2017) *corrigendum*	Excluded (No diagnosis of AD or MCI likely due to AD)		
	29789032	Amanzio et al. (2018)	Included	Neuropsychological assessment	Anosognosia Questionnaire for Dementia (AQ-D, Migliorelli et al., 1995)
	30531365	De Feis et al. (2019)	Included	Neuropsychological assessment	Patients and caregivers report of symptoms
Frontotemporal dementia	27534380 28633865	Amanzio et al. (2016)Amanzio et al. (2017) *corrigendum*	Included	Structural MRI and neuropsychological assessment	Anosognosia Questionnaire for Dementia (AQ-D, Migliorelli et al., 1995)
Acquired brain injury	23962086	Palermo et al. (2014)	Included	Functional MRI and neuropsychological assessment	Anosognosia Questionnaire for Dementia (AQ-D, Migliorelli et al., 1995)

*The characteristics of the original articles (considering etiopathogenesis, methods, and awareness assessment scales) are presented. AD, Alzheimer’s disease; MCI, mild cognitive impairment; MRI, magnetic resonance imaging.*

Only eleven articles suited criteria for this mini-review. They will be described according to the theoretical framework previously introduced.

## “Reduced Awareness” [and] “Alzheimer”

Among the articles selected on the AD, from the first published to the most recent, there are those of Bonney et al. (2007), Orfei et al. (2010), Amanzio et al. (2011, 2013), Galeone et al. (2011), and Spalletta et al. (2014), another one by Amanzio et al. (2018) and De Feis et al. (2019).

Bonney et al. (2007) analysed how a reduced awareness of executive dysfunction, characterising a “dysexecutive syndrome” in 24 participants with mild AD, may be related with their caregivers’ burden. In line with their hypotheses, the authors observed an association between caregiver’s burden and reduced awareness of deficits related to executive dysfunction, suggesting that early detection of executive dysfunctions may help develop effective strategies to reduce the care burden.

In order to analyse awareness of illness, Orfei et al. (2010) recruited 38 mild AD patients, 35 amnesic mild cognitive impairment (a-MCI) and 38 multiple domain MCI (md-MCI) subjects. Results showed that patients with mild AD were more anosognosic than both MCI groups, and md-MCI subjects presented a reduced awareness of their illness. The authors pointed out that a reduced awareness of illness should be studied along with anosognosia in AD. Moreover, anosognosia in mild AD was associated to increased age and reduced basic ADL autonomy, while verbal episodic memory deficits were correlated with decreased awareness of cognitive impairment only in a-MCI patients.

Galeone et al. (2011) investigated a reduced awareness of memory deficits in 25 a-MCI and 15 mild AD patients, pointing out that both groups overestimated their memory performances. In particular, subjects presented decreased awareness for memory deficit and memory-monitoring difficulties, associated with executive functioning. The authors demonstrated that a reduced awareness could characterise even early stages of AD, such as a-MCI subjects.

Spalletta et al. (2014) analysed the neuroanatomical correlates of awareness of illness in 36 a-MCI patients, followed for five years, in order to understand whether they could be considered risk factors for conversion to AD. The authors reported that converter subjects showed a greater reduction of self-awareness of memory deficits, which correlated with reduced grey matter volume of the ACC and of the inferior frontal cortex. Their results highlight how the awareness of deficit in converter and non-converter aMCI patients is characterised by different pathogenic mechanisms. In particular, converter subjects showed a dysregulation of the cognitive control, such as selection, manipulation and inhibition of self-information. The authors concluded that these pathogenic mechanisms, related with augmented risk of AD conversion, could also support reduced self-awareness in other neurological conditions.

More recently, De Feis et al. (2019) investigated the relationship between a reduced awareness of memory deficits and the need for in-home assistance in 192 patients with probable AD and Lewy Bodies Dementia. The authors reported that a reduced self-awareness of memory deficits could be associated with a more frequent use of home health care services. These results are important as they might have clinical, caregivers, and health care implications.

Finally, Amanzio et al. (2018) analysed the association between reduced awareness and executive dysfunction in 144 patients with different cognitive deficits –from “Mild Cognitive Impairment (MCI) likely due to AD” to “mild AD patients.” As baseline executive dysfunction predicts worsening of IADL over time and progression to AD, results showed that executive dysfunction, associated with reduced IADL awareness, were selectively characterised by a worst performance on response inhibition, self-monitoring and set-shifting tasks (Amanzio et al., 2018).

In their other studies, considered in this mini-review, Amanzio et al. (2011, 2013) underlined the importance of executive dysfunction related with MPFC and impaired self-awareness in AD. In particular, the authors (Amanzio et al., 2013) estimated the role of different cognitive and mood changes variables taking into consideration 117 AD patients. Results showed that inhibition, self-monitoring and set-shifting were associated with awareness of iADL. Moreover, a tendency to hypomania and apathy seemed related to reduced behavioural awareness. Amanzio et al. (2011) also evaluated the neural underpinnings of reduced self-awareness in 29 AD patients, focusing on MPFC and anterior cingulate cortex functionality. Unaware patients showed a more evident reduction of activity of the right anterior cingulate area and of the rostral prefrontal cortex, a higher dysfunction of the MPFC, in particular in the dorsal division of the ACC (MCC), and in heteromodal association areas. In addition, they showed an hypofunctionality of the right post-central gyrus, of the associative cortical areas, such as the right parieto-temporal-occipital junction and the left temporal gyrus, of the striatum, and of the cerebellum.

These results show that reduced awareness of deficits during the first phases of AD is related to an hypoactivity of the cingulo-frontal and parieto-temporal regions and, on the behavioural side, to apathy and disinhibition (Amanzio et al., 2011).

## “Reduced Awareness” [and] “Frontotemporal Dementia”

The only works concerning the reduction of awareness in FTD patients, identified through the research strategy, are the original article by Amanzio et al. (2016, 2017). The authors explored primarily the anatomo-functional brain changes related to IADL in 67 bvFTD patients and, secondly, the neural correlates of reduced awareness in the IADL domain.

The Anosognosia Questionnaire for Dementia (AQ-D: Migliorelli et al., 1995) was used to assess the presence of reduced awareness for the instrumental domain (AQD_iADL).

The authors found disabilities in IADL and a reduced AQD_iADL to be associated with atrophy of the medial prefrontal cortex, in which the mid-cingulate cortex, the anterior dorsal cortex, cuneus and insula played an important role (Amanzio et al., 2016, 2017). The neurocognitive approach applied to bvFTD proves effectiveness in illustrating the association between brain pathology and cognitive and behavioural deficits (McGlynn and Schacter, 1989; Lezak et al., 2004).

## “Reduced Awareness” [and] “Acquired Brain Injury”

Palermo et al. (2014) reported a clinical description of a self-unaware patient with an ischemic injury in the right ACC. In their study, the only one present in literature on this topic, the authors suggested that the damage in the cingulo-frontal region could be considered as one of the neurobiological substrates of the persistent reduced self-awareness of the patient. In patients with ABI, the association between executive functions and self-awareness is characterised by deficits in: response inhibition abilities, mental flexibility (Burgess et al., 1998; Trudel et al., 1998), self-regulation of errors (Burgess et al., 1998; Ownsworth and Fleming, 2005), self-monitoring of action performance with the impact of error (in terms of online awareness), and updating self-information about errors (Vuilleumier, 2004; Ownsworth et al., 2008). Thus, measures for self-regulating errors provide a method that can be useful to examine the contribution of neuropsychological factors in awareness deficits, mainly focusing on the role of MPFC in ABI patients (Amodio and Frith, 2006; Ownsworth et al., 2007). Indeed, MPFC and the cingulate cortex are considered primary areas for self-awareness (Johnson et al., 2002).

## Reduced Awareness and Other Neurodegenerative Disorders: Parkinson’s Disease (PD)

Considering PD, the presence of dyskinesias-reduced-self-awareness (DRSA) had been related to executive and metacognitive impairments and, apparently, it arose because of the dopaminergic overstimulation of the mesocorticolimbic areas (Amanzio et al., 2010, 2014; Palermo et al., 2017). In addition, a relationship between DRSA and an hypoactivity of the bilateral ACC, bilateral anterior insular cortex and right dorsolateral prefrontal cortex had been showed (Palermo et al., 2018). These results indicate how the executive deficits impact on reduced self-awareness in neurodegenerative disorders, and how the ACC is the main hub of the damaged response-inhibition circuit.

## Clinical Implications and Recommended Good Practices

A detailed neuropsychological assessment that includes investigation of possible self-awareness disturbances–and its behavioural sequel–across a wide range of domains, should be set in AD, bvFTD and ABI.

Specific executive-metacognitive functions, often associated with the presence of self-awareness reduction, should be accurately studied. When cognitive and functional changes occur, it seems that metacognitive functioning plays an important role in modifying the approach to everyday activities. Indeed, a reduction in IADL self-awareness should also be taken in great consideration.

Moreover, neuroimaging assessment should be implemented both from a functional and structural point of view to better outline DLPFC-ACC system dysfunction, tapping cognitive-action-control, that may cause reduced self-awareness disorders (Amanzio et al., 2011; Palermo et al., 2015, 2018).

Self-awareness disorders can lead to poor adherence to pharmacological treatment and prognosis in patients with neurological diseases (Acharya and Sánchez-Manso, 2020). Therefore, frailty determinants and psychosocial factors should also be assessed in the context of a neurocognitive perspective, being essential variables to identify vulnerable subjects needing further support (Amanzio et al., 2016).

## Limitation of Studies

The studies described in this mini-review present some limitations. Additional studies are needed to better evaluate the appearance of self-awareness reductions throughout the duration of the disease, to better understand associations with executive and meta-cognitive domains also in subjects with MCI. Particularly, longitudinal studies are required to better monitor neurological patients with a reduced self-awareness at different transition points, defining specific primary and secondary prevention assessments. The proposed evaluation approach should lead to a careful development of tailored longitudinal interventions for patients, and guidance for health professionals to maximise prognosis and quality of life.

## Future Research Perspective

Programs enhancing executive-metacognitive functions should be implemented to promote self-awareness in individuals with neurological disorders and cognitive impairment. Toglia et al. (2010) had previously showed how metacognitive strategy training could be useful for ameliorate self-awareness. In fact, it seems that a punctual self-awareness assessment and intervention can assist in enabling better and earlier patients at risk of poor treatment response. Moreover, clinicians may improve adherence to treatments using the proper strategies of engagement.

## Conclusion

Metacognitive executive dysfunction and MPFC impairment, delineated through the neurocognitive model, may help to understand how the central executive system could contribute to self-awareness disorders related to AD, bvFTD and ABI (Starkstein et al., 1995; Litvan et al., 1996, 1997; Agnew and Morris, 1998; Amanzio et al., 2011, 2013, 2016; Palermo et al., 2014). The similarity of the neuropsychological profile, in terms of overlapping the symptoms associated with the onset of self-awareness, seems to authorise a transposition of the interpretative model in different neurological disorders (Palermo et al., 2014).

Conscious experience of post-injury/neurodegenerative disease changes requires an interaction among relevant functional domains, comparator mechanisms within the central executive system to detect deficits, and the CAM. The studies presented highlight an association between reduced self-awareness and executive dysfunction related with MPFC anatomo-functional impairment, causing difficulties in response-inhibition, cognitive set-shifting and action-monitoring performances. Results suggested that a selective MCC lesion might be associated with reduced self-awareness, which could remain over time even in a context of partial recovery of cognitive functions different from the executive ones.

This mini-review results support the explanatory effectiveness of the CAM theoretical model (Agnew and Morris, 1998; Amanzio et al., 2013; Mograbi and Morris, 2014), for which damage to the “comparator mechanisms” in the executive system compromises the capacity to update the “personal database” with current information about themselves, sometimes referred to as «developing a “petrified-self”» (Mograbi et al., 2009; Steward et al., 2019).

## Author Contributions

MA conceived the content of the review, draft the manuscript and supervised changes, took part in critique processes. Moreover, she updated the theoretical model of self-awareness considering executive-metacognitive functions. MB participated in the drafting of the manuscript, took part in the review and critique process. GC participated in the drafting of the manuscript. SP wrote the second version of the manuscript, produced infographics, took part in the review and critique processes. All authors contributed to the article and approved the submitted version.

## Conflict of Interest

The authors declare that the research was conducted in the absence of any commercial or financial relationships that could be construed as a potential conflict of interest.
